# Clinical outcomes of stereotactic magnetic resonance image‐guided adaptive radiotherapy for primary and metastatic tumors in the abdomen and pelvis

**DOI:** 10.1002/cam4.4139

**Published:** 2021-07-20

**Authors:** Stephanie M. Yoon, Elaine Luterstein, Fang‐I Chu, Minsong Cao, James Lamb, Nzhde Agazaryan, Daniel Low, Ann Raldow, Michael L. Steinberg, Percy Lee

**Affiliations:** ^1^ Department of Radiation Oncology University of California Los Angeles Los Angeles CA USA; ^2^ University of California San Diego School of Medicine San Diego CA USA; ^3^ Department of Radiation Oncology MD Anderson Cancer Center University of Texas Houston TX USA

**Keywords:** abdominal pelvic tumors, cancer management, MR‐guided radiation therapy, stereotactic ablative radiotherapy, stereotactic body radiation therapy

## Abstract

**Purpose:**

Stereotactic body radiotherapy (SBRT) delivers ablative doses with excellent local control. However, implementing SBRT for abdominal and pelvic tumors has been limited by the risk for treatment‐related gastrointestinal toxicity. MRI‐guided radiotherapy may ameliorate these risks and increase the therapeutic ratio. We report the clinical outcomes of stereotactic MRI‐guided adaptive radiotherapy (SMART) for primary and metastatic tumors in the abdomen and pelvis.

**Methods:**

From November 2014 to August 2017, the first 106 consecutive patients with 121 tumors in the abdomen and pelvis were treated with SMART at a single institution. Of the cohort, 41.5%, 15.1%, and 43.4% had primary, locally recurrent, and oligometastatic tumors, respectively. SMART was delivered using a tri‐cobalt‐60 gantry with on‐board 0.35 Tesla MRI with respiratory breath‐hold and daily adaptive re‐planning when anatomically necessary. A median of 40Gy in five fractions was prescribed. The Common Terminology Criteria for Adverse Events v.4.03 was used to score treatment‐related toxicities. Local control (LC), progression‐free survival (PFS), and overall survival (OS) were estimated using Kaplan–Meier method.

**Results:**

Of the 510 treatments, seventy‐one (13.9%) were adapted. Fatigue, nausea, and pain were the most common acute toxicities. 0.9 and 0% of patients experienced acute grade three and four toxicities, respectively. 5.2 and 2.1% of patients experienced late grade three and four toxicities, respectively. After a median follow‐up of 20.4 months, the 2‐year LC rate was 74% on a per‐lesion basis. Two‐year LC was 96% for lesions that were treated with BED_10_≥100 versus 69% for BED_10_<100 (*p* = 0.02). PFS was significantly different between patients with and without locally controlled tumors (2‐year PFS 21 vs. 8%, *p* = 0.03). Two‐year OS was 57% for the entire cohort.

**Conclusions:**

Favorable LC and PFS outcomes were observed with minimal morbidity for tumors in the abdomen and pelvis treated with SMART. Future prospective clinical trials to validate these findings are warranted.

## Graphical abstract

Implementing stereotactic body radiotherapy for abdominal and pelvic tumors has been limited by risk for treatment‐related gastrointestinal toxicity. In the largest reported clinical series to date of stereotactic magnetic resonance image‐guided adaptive radiotherapy, favorable local control and progression‐free survival were observed with minimal morbidity.

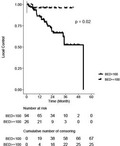

## 1 INTRODUCTION

Stereotactic body radiotherapy (SBRT) is an increasingly utilized technique that delivers ablative radiation doses to tumors while minimizing radiation exposure to surrounding healthy tissues.^1^ Compared to doses associated with conventional radiation techniques, ablative doses disrupt cellular function and mitosis which translates to improved oncologic outcomes such as local control and survival for several malignancies.^2^, ^3^, ^4^ SBRT requires accurate evaluation of tumor position; however, implementing it more broadly for abdominal and pelvic tumors is limited by the risk of injuring serially functioning normal tissues such as the stomach, duodenum, and small bowel.^5^, ^6^, ^7^, ^8^

Tumors in the abdomen/pelvis are dynamic, exhibiting positional changes relative to organs‐at‐risk (OARs) due to respiratory motion, peristalsis, and intrinsic deformation.^9^, ^10^, ^11^ Minimizing positional and anatomic uncertainties with computed tomography (CT) image guidance has provided a stepwise improvement in tumor localization and radiation delivery. However, current capabilities with CT guidance have limited soft‐tissue contrast, are susceptible to motion degradation, and depend on surrogate tracking like fiducial markers.^12^

Magnetic resonance image‐guided radiotherapy (MRgRT) can ameliorate several challenges when delivering radiation to the abdomen and pelvis.^13^ It provides the ability to image patients before and during radiotherapy with enhanced soft‐tissue contrast, allowing for more accurate tumor localization. Online adaptive radiotherapy (ART), gating with real‐time tumor tracking, and breath‐hold techniques further minimize uncertainties surrounding tumor position and improve accuracy of radiation delivery. Altogether, MRgRT holds a high potential to safely escalate dose to targets that are in close proximity to critical neighboring organs. In several series across multiple gastrointestinal sites, escalating biologic effective dose (BED), particularly beyond 100Gy, has been associated with improvement in local control and overall survival.^14^, ^15^, ^16^, ^17^

MRgRT has shown to be safe and technically feasible from early institutional experiences.^18^, ^19^, ^20^ Emerging data suggest that dosimetric gains with stereotactic MRI‐guided adaptive radiotherapy (SMART) have correlated with encouraging clinical outcomes; however, literature remains sparse especially outside of clinical trials.^15^, ^21^, ^22^ As SMART has become increasingly adopted into clinical practice, we report the safety, efficacy, and its utilization in the largest cohort of patients with primary and metastatic tumors in the abdomen and pelvis.

## 2 MATERIALS AND METHODS

### 2.1 Study population

A retrospective study of the first 106 consecutive patients treated with SMART from November 2014 to August 2017 at a single institution was conducted. All patients had either medically inoperable tumors or oligometastatic disease, defined as involving less than or equal to five disease sites, and were clinically and technically eligible for SBRT. Seventeen of the included patients were enrolled onto a prospective trial evaluating SMART for primary or metastatic hepatic malignancies. Patients were not offered SMART if they had contraindications toward MR imaging, had prior radiotherapy in or near the anticipated treatment field, were pregnant, or had concurrent medical illnesses that precluded them from completing radiotherapy treatments. Thirteen patients underwent SMART to multiple tumors. The dominant tumor, which underwent the earliest treatment, was considered in per‐patient analyses while all tumors were considered in per‐lesion analyses.

### 2.2 CT/MRI simulations and initial treatment planning

Simulation, initial treatment planning, MRgRT platform, and treatment planning system were previously described.^23^, ^24^ SMART was delivered using MRIdian (ViewRay Inc., Sunnyvale, CA) consisting of a tri‐cobalt‐60 ringed gantry with on‐board 0.35 T MRI imaging. Prescription dose was chosen by treating physicians based on several factors including anatomic location, tumor histology, institutional or national guidelines, quality assurance, and experience. The coverage goal was generally at least 95% planning target volume (PTV) to receive at least 95% of the prescription dose (V95% > 95%) or at least 95% of the gross target volume (GTV) to receive at least 100% of the prescription dose (V100% > 95%). Strict OAR dosimetric constraints were the volume to stomach, duodenum, and small bowel receiving 35Gy or greater to be less than 0.5mL (V35Gy < 0.5 ml), and for the maximum dose to the spinal cord to be less than 30Gy (Dmax < 30Gy). All other OAR constraints outlined by the American Association of Physicists in Medicine (AAPM) TG‐101 were followed.^25^

### 2.3 Online adaptation and treatment delivery

Details regarding online adaptive re‐planning and treatment delivery were previously published.^26^, ^27^, ^28^ Daily volumetric breath‐hold MR scan was taken with patients in the treatment position prior to each fraction utilizing the same protocol as during simulation. Contours of the GTV from the original or prior fraction’s plan were transferred and rigidly registered with alignment based on soft tissue. When applicable, a PTV was generated by adding a 5 mm isotropic margin to the breath‐hold GTV. Critical OAR contours were auto‐generated and manually edited by an adaptive planner. The predicted dose based on the daily volumetric MR scan was recalculated using the electron density image deformed from CT treatment planning. The managing physician evaluated the predicted plan based on pre‐set dosimetric criteria defined by the treating physician.^26^ If the predicted plan violated dosimetric criteria by exceeding strict OAR constraints (outlined in the previous section) or undercovering the PTV or GTV to less than 90% of the volume, an online adaptive plan was generated. Additional dose constraints defined by treating physicians were provided to managing physicians to guide the decision to adapt. Priority of these constraints varied depending on tumor location and dose fractionation regimen. Dosimetry of the adaptive plan was compared against the original plan, and the superior plan was delivered. Online quality assurance was performed prior to treatment delivery. Respiratory breath‐hold techniques were used for the majority of patients. Breath‐hold‐based gating mitigated intrafractional motion throughout the radiation delivery. Details on gating parameters were previously published.^29^ Based on the performance of the gating system evaluated by a respiratory motion phantom and radiochromic film, generating a gating boundary (defined as a 3 mm expansion margin to the GTV from the volumetric MRI scan) and allowing up to 10% excursions of the GTV beyond this boundary could compensate for respiratory motion. GTV excursions of more than 10% paused the radiation beam.^29^

### 2.4 Follow‐up, data collection, and statistical analysis

Institutional board review approval was in place for this investigation. Relevant patient demographic, clinical, and treatment data were prospectively encoded into a department registry. Patients were regularly seen in clinic to evaluate for the treatment response and radiation‐associated toxicities with CT abdomen/pelvis scans. Treatment response was assessed by the Response Evaluation Criteria in Solid Tumors (RECIST), with tumor LC defined as stable disease, partial response, or complete response.^30^ Growth of treated tumor compared to prior CT scans was considered as local failure. The Common Terminology Criteria for Adverse Events (CTCAE) version 4.03 was used to score treatment‐related toxicities. Toxicities were acute when occurring within 90 days from completion of SMART, and any afterward was considered late. A CTCAE toxicity score of three or greater was considered severe.

Descriptive statistics summarized patient demographics, clinical and baseline treatment features, and toxicity. Chi‐square test or Fisher’s exact test, when applicable, assessed the association between incidence of toxicities by grade and biologic effective dose (BED_10_) groups, with <100Gy defined as low‐dose group and ≥100Gy defined as high‐dose group. Time‐to‐event analysis was performed with the primary endpoint defined as time from the start of radiotherapy to occurrence of event. Endpoints of interest were time to local failure, time to disease progression or death, and time to death. Tumor LC, progression‐free survival (PFS), and overall survival (OS), respectively, were estimated and compared via Kaplan–Meier method and log‐rank test. Multivariable Cox proportional hazards (PH) and Fine Gray competing risk (with death considered as the competing event) regression evaluated the relationship between time to local failure and covariates including tumor diameter, location, elapsed treatment days, BED_10,_ and prior therapies (surgery, radiation, and ablations) with random effects to account for within‐subject correlation. The overall significance of the categorical variable within multivariable Cox PH regression model was assessed by the likelihood ratio test (LRT). The PH assumption was examined for Cox PH model via the diagnostic plot of Schoenfeld residuals. Significance level was set at 5% and all test were two‐sided. All analyses were conducted using R 3.6.0 (R Foundation for Statistical Computing) with packages *survival, survminer, coxme, and crrSC*.^31^, ^32^, ^33^, ^34^, ^35^

## 3 RESULTS

Demographic and tumor characteristics are summarized in Table 1. On a per‐patient basis, 44 (41.5%) patients had medically inoperable primary, 16 (15.1%) had locally recurrent, and 46 (43.4%) had oligometastatic tumors. Most common histologies were pancreatic cancer (25, 23.6%), cholangiocarcinoma (16, 15.1%), and hepatocellular carcinoma (11, 10.4%). On a per‐lesion basis, majority of tumors resided in the liver (46, 38.0%) and pancreas (26, 21.5%). More specifically, 22 of 46 (47.8%) liver tumors were centrally located near critical normal tissues. Average tumor diameter was 3.0cm (SD 1.7). Median prescription dose was 40Gy (range 24–60) in five fractions (range 3–5), corresponding to a median BED_10_ of 72Gy (range 37.5–180.0). Eighty‐four (79.2%) patients were in the low‐dose BED group and 22 (20.8%) patients were in the high‐dose BED group. At least one patient in the high‐dose group was treated for multiple tumors. Notably, 22 of 27 tumors (81.5%) tumors treated to BED_10_ ≥100 were hepatic. Five hundred and ten treatment fractions were delivered. Seventy‐one fractions (13.9%) from 28 patients (26.4%) underwent online adaptation to avoid OAR overdose or GTV undercoverage. Forty‐nine (75.4%) of adapted fractions occurred for pancreatic tumors.

**TABLE 1:** Demographic and tumor characteristics

Age, mean (*SD*)	65 (12.6)
Gender, *n* (%)	
Female	56 (52.8%)
Male	50 (47.2%)
Karnofsky Performance Status, *n* (%)	
100	13 (12.3%)
90	52 (49.1%)
80	23 (21.7%)
70	6 (5.7%)
60	2 (1.9%)
Unknown	10 (9.4%)
Histologic diagnosis, *n* (%)	
Pancreatic cancer	25 (23.6%)
Cholangiocarcinoma	16 (15.1%)
Hepatocellular carcinoma	11 (10.4%)
Ovarian cancer	9 (8.5%)
Prostate cancer	8 (7.6%)
Other	37 (34.9%)
Radiation treatment setting, *n* (%)	
Primary	44 (41.5%)
Locally recurrent	16 (15.1%)
Oligometastatic	46 (43.4%)
Prior therapies, *n* (%)	
Surgery	56 (52.8%)
Radiation	37 (34.9%)
Ablation	15 (14.2%)
Treatment sites^†^, *n* (%)	
Liver	46 (38.0%)
Central	22 (47.8%)
Peripheral	24 (52.2%)
Pancreas	26 (21.5%)
Hepatobiliary	7 (5.8%)
Adrenal glands	7 (5.8%)
Prostate/ Prostate bed	6 (5.0%)
Pelvic side wall	6 (5.0%)
Other	22 (18.2%)
Target diameter (cm)^†^, mean (SD)	3.0 (1.7)
Total radiation dose^†^, median (range)	40 (24–60)
Treatment fraction^†^, median (range)	5 (3–5)
BED_10_ (Gy)^†^, median (range)	72 (37.5–180)

Abbreviations: BED, biologic effective dose; *SD*, standard deviation.

† Per‐lesion basis (*n* = 121). All other results are per‐patient basis (*n* = 106).

Median follow‐up for the cohort was 20.4 months (range 0.4–52.2), and were 21.3 (range 1.2–52.2) and 18.2 months (range 0.4–43.0) for patients in the low‐ and high‐dose BED groups, respectively. Table 2 summarizes acute and late treatment‐related toxicities. Sixty‐nine (65.1%) patients experienced grade one or two acute GI toxicity. Only one (0.9%) patient developed an acute grade three duodenal ulcer with perforation just under 3 months after completing the treatment. This patient was treated for an intrahepatic tumor early in the study period, and 3.83 ml of stomach received ≥35Gy. From this experience, strict institutional constraints for stomach and bowel were established (V35Gy < 0.5 ml), and severe acute toxicities were not further observed. No patients developed acute grade four or five toxicities. Most patients experienced nausea (25, 23.6%), fatigue (23, 21.7%), and pain (17,16.0%). No association between acute toxicities by grade and BED dose groups was found.

**TABLE 2:** Summary of acute and late toxicities

Acute toxicity	Entire cohort, *n*(%)		BED < 100, *n*(%)	BED ≥ 100, *n*(%)	*p* value*
Total patients		106	84	22	
Missing patients		0	0	0	
Grade					
1		60 (56.6%)	47 (56.0%)	13 (59.1%)	0.98
2		9 (8.5%)	8 (9.5%)	1 (4.5%)	0.68
3		1 (0.9%)	1 (1.2%)	0 (0%)	1.00
4		0 (0%)	0 (0%)	0 (0%)	NA
Late Toxicity					
Total patients		97	77	20	
Missing patients		9	7	2	
Grade					
1		8 (8.2%)	7 (9.1%)	1 (5.0%)	1.00
2		5 (5.2%)	5 (6.5%)	0 (0%)	0.58
3		5 (5.2%)	4 (5.2%)	1 (5.0%)	1.00
4		2 (2.1%)	1 (1.3%)	1 (5.0%)	0.37

Abbreviation: BED, biologic effective dose.

* *p* values are obtained using Chi‐squared test or Fisher’s exact test when appropriate.

Of 97 patients with available late toxicity data, 13 (13.4%) patients developed late grade one or two toxicities. Five (5.2%) patients developed late grade three toxicities, four of whom were in the low‐dose group. Two (2.1%) patients exhibited late grade four toxicities. One patient developed sepsis from a peri‐rectal abscess after treatment to a rectal mass (BED_10_ < 100). The peri‐rectal abscess appeared 30 months after treatment within the 50% isodose region. Another patient developed sepsis from a peri‐biliary abscess 27 months after completing treatment to central liver tumor (BED_10_ ≥100). This abscess also occurred within the 50% isodose region. No patient experienced late grade five toxicity, and no association between late toxicities by grade and BED dose groups was found.

Twenty‐eight of 121 tumors (23.1%) developed local failure. Among the most common sites of failure were the pancreas/pancreatic bed (9, 32.1%) and liver (6, 21.4%), which were initially treated to median BED_10_ = 72Gy (range 72.0–72.0) and 64.8Gy (range 51.3–72.0), respectively. Twenty‐seven of 28 (96.4%) locally failed tumors occurred in the low‐dose BED group while only one (3.6%) in the high‐dose BED group failed. Online ART was employed for eight of these recurrent tumors (all located in pancreas), and dose could not be escalated without violating OAR constraints. The remaining 20 recurrent tumors were treated without adaptation. Although multivariable regression analysis had a tendency to be unstable or, increase in BED_10_ or having prior radiation had lower risk of local failure by Cox PH regression with random effects (HR 0.94, 95% CI 0.89–0.98, *p <* 0.01; HR 0.16, 95% CI 0.04–0.66, *p =* 0.01, respectively). Adrenal tumors had higher risk to local failure relative to liver tumors by Cox PH regression (HR 34.16, 95% CI 3.30–353.48, *p <* 0.01), but tumor location was not found to be an overall significant predictor for local failure by LRT (*p* *= *0.06). Exploratory analysis of the seven treated adrenal tumors found that two tumors failed locally, one receiving BED_10_ = 72Gy and the other 100Gy. All adrenal tumors were treated with 3–5 mm margin from the GTV with breath‐hold gating.

**FIGURE 1:**
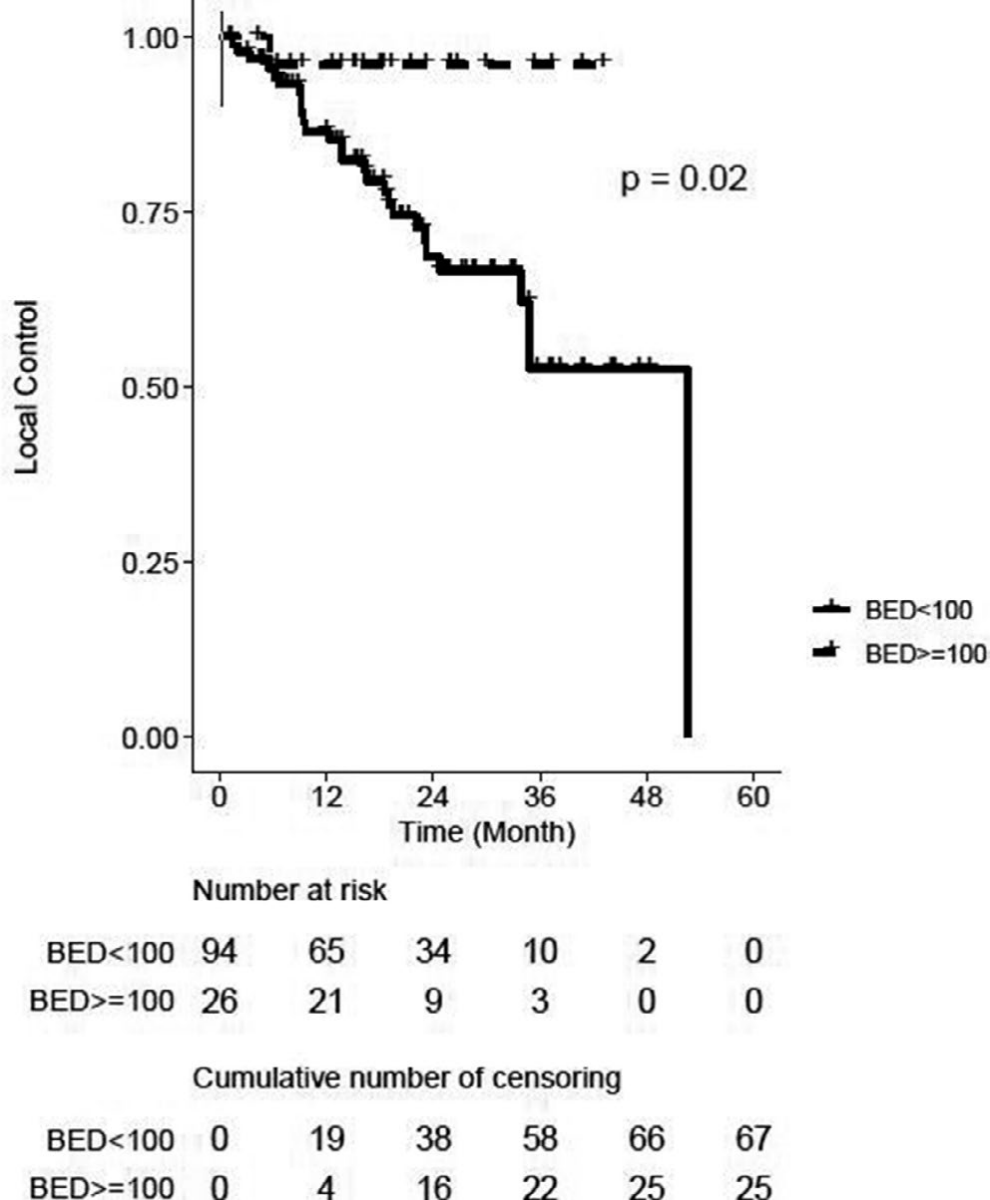
Kaplan–Meier curve for local control, stratified by biologic effective dose (BED10) groups. ^+^Censored cases. ^†^Per‐lesion analysis

On a per‐patient basis, 1‐ and 2‐year LC rates were 87% (95% CI 78–92) and 71% (95% CI 59–80), respectively. On a per‐lesion basis, LC rates for the cohort were 89% (95% CI, 81–93) and 74% (95% CI, 63–82) at 1 and 2 years, respectively. Figure 1 shows LC at 2 years was 69% and 96% for tumors in the low‐ and high‐dose BED groups, respectively (*p* = 0.02).

**FIGURE 2:**
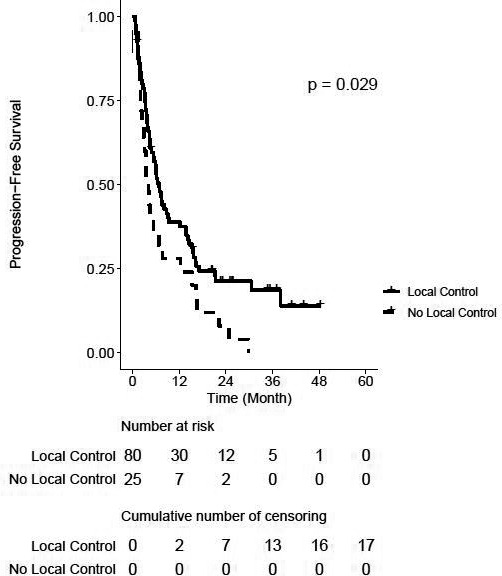
Kaplan–Meier curve for progression‐free survival, stratified by tumor local control. ^+^Censored cases

Seventy‐nine (74.5%) patients developed disease outside the irradiated area. PFS at 1 and 2 years was 36% (95% CI, 27–45) and 18% (95% CI, 11–26) for the entire cohort. Two‐year PFS among patients with tumor LC was 21% compared to 8% among patients with local failure (*p* = 0.03) (Figure 2). Fifty‐two (49.1%) of patients had died at last follow‐up. OS rates at 1 and 2 years for the entire cohort were 79% (95% CI, 70–86) and 57% (95% CI, 46–66), respectively. OS was not significantly different between patients who did and did not exhibit tumor LC (2‐year OS 60 vs. 48%, *p* = 0.10) or between high‐ and low‐dose groups (2‐year OS 49 vs. 59%, *p* = 0.98).

## 4 DISCUSSION

In this investigation, we report the largest clinical experience of SMART for abdominal and pelvic tumors. We delivered median BED_10_ of 72Gy, with 22.3% of tumors receiving BED_10_ ≥100. In spite of such dose escalation, only one (0.9%) patient experienced a severe acute toxicity (grade 3), and seven (7.3%) patients experienced severe late toxicities. More striking is that no association between toxicity incidences by grade and BED groups was found. Safe dose escalation corresponded with favorable 1‐ and 2‐year LC rates of 89 and 74%, respectively, with tumors receiving BED_10_ ≥ 100 experiencing higher LC. Moreover, patients whose tumor was locally controlled experienced favorable 2‐year PFS. Altogether, we found that SMART was associated with encouraging LC and PFS outcomes with minimal morbidity.

Early attempts with SBRT to various abdominal tumors demonstrated significant and severe GI toxicities.^6^, ^36^, ^37^, ^38^, ^39^, ^40^ For example, Schellenberg et al. reported acute and late grade two or greater toxicities in 18.8 and 43.8% of patients with locally advanced pancreatic cancer who had been treated to 25Gy in a single fraction.^36^ In the oligometastatic setting, a phase two prospective trial reported a 20% absolute increase in grade two or higher acute toxicities when all sites of disease were treated with SBRT compared to the standard of care, and 5% of patients receiving SBRT experienced treatment‐related deaths.^41^

The low incidences of severe toxicities observed in this study despite delivering high doses are consistent with clinical experiences with SMART. In a phase one trial of SMART for abdominal and pelvic tumors, no patients developed severe acute toxicities and only one (5%) patient developed an asymptomatic grade two gastric antrum ulcer.^19^ Table 3 summarizes toxicities from key selected studies of SMART. Improved image quality and real‐time tumor surveillance in relation to surrounding normal tissues with MR guidance have afforded radiation oncologists to treat with smaller margins, potentially allowing delivery of ablative doses without worsening toxicity or clinical outcomes.^15^, ^29^, ^42^ Currently, accurate cumulative dose calculations are not available on commercial MRgRT platforms. Since critical tissues are deformable and mobile, the cumulative maximum radiation exposure to a particular area of tissue could be less than previously assumed; therefore, dose constraints used during treatment planning and online ART may be conservative. Monitoring critical normal structures in real time and recalculating dose with MR guidance provides an opportunity to understand the true tolerance of abdominal and pelvic organs to radiation and is a direction of future research.

**TABLE 3:** Summary of selected clinical reports of stereotactic magnetic resonance adaptive radiation therapy (SMART) for tumors in the abdomen and pelvis

Study	Median f/u (months)	Patients	Treatment site	Treatment regimen	Median BED_10_	Local control	Overall survival	Severe^†^ acute toxicity	Severe^†^ late toxicity
Rosenberg et al.^42^	21.2	26	Liver	50Gy (range 30–60) in 5 fx (range 6–12)	100	80.4% (at median f/u)	1 year 69%, 2 year 60%	NA	7.7% (at median f/u)
Henke et al.^18^	15	20	Various abdominal sites	50Gy (range 50–60) in 5 fx (range 4–5)	100	90% (at median f/u)	1 year 75%	0%	0%
Luterstein et al.^43^	15.8	17	Hepatobiliary	40Gy (40–50) in 5 fx (range 3–5)	72	1 year 85.6%, 2 year 73.3%	1 year 76%, 2 year 46.1%	6%	0%
Bruynzeel et al.^22^	1.5	101	Prostate (localized)	36.25Gy in 5 fx	62.5	NA	NA	GU: 5.9% GI: 0%	NA

Abbreviations: f/u, follow‐up; Fx, fraction; GI, gastrointestinal; GU, genitourinary; NA, not available.

† Severe defined as CTCAE grade three or higher.

Emerging experiences with SMART or MRgRT have also reported encouraging clinical outcomes. A phase one SMART trial reported 2 of 20 (10%) patients experiencing local tumor progression following treatment with 50Gy in five fractions (BED_10_ = 100) to abdominal/pelvic malignancies and 1‐year OS of 75%.^19^ In a retrospective series of unresectable locally advanced cholangiocarcinoma, the 2‐year LC rate and OS were 73.3% and 46.1% respectively, following SMART to 40Gy in 5 fractions (BED_10_ = 72).^43^ In a multi‐institutional review of patients with medically inoperable pancreatic cancer, 2‐year OS for high‐dose radiotherapy (defined as BED_10_ ≥ 70) was 49 vs. 30% for standard dose radiotherapy (BED_10_ < 70) (*p* = 0.03) without the development of severe GI toxicities.^15^ Almost all pancreatic tumors in our cohort received BED_10_ = 72 without worsening toxicity which improves upon current CT‐guided SBRT prescription doses of 25–33Gy in three to five fractions (BED_10_ = 45.8–54.8) in order to ensure safety.^40^

In a multi‐institutional series of primary or metastatic liver tumors, freedom from local progression was 80.4% after median dose of 50Gy in five fractions (BED_10_ = 100).^42^ Two‐year OS in this cohort was 60%, with 7.7% of patients experiencing acute grade three GI toxicity. With prospective trials beginning to show improvement in clinical outcomes after metastasis‐directed therapy, more patients will likely undergo SBRT for oligometastatic disease in the near future.^4^, ^44^ SMART is a therapeutic option that could mitigate potential severe toxicities and improve clinical outcomes for tumors located in challenging locations in the abdomen and pelvis.^4^

Higher BED was associated with improved LC in our study. Interestingly, 27 of the 28 tumors that failed locally received a BED of less than 100Gy, and higher BED had lower risk to local failure on multivariable analysis. These findings align with evidence from several disease sites that reported a critical BED of 100Gy was associated with improved durable LC and survival outcomes.^14^, ^16^, ^17^, ^28^, ^45^ However, delivering BED_10_ ≥ 100 was possible primarily for liver tumors in our cohort which has also been achieved with SBRT under CT guidance.^46^, ^47^, ^48^ All liver tumors that locally failed in this study were treated to median BED_10_ of 64.8 which was low compared to the dose often associated with improved LC.^46^, ^47^, ^48^

Dose escalation to BED_10_ ≥100 may not be possible for select disease locations. In particular, local failures still occurred in the pancreas even after delivering median BED_10_ = 72. In eight locally failed pancreatic tumors, further dose escalation was not possible without exceeding OAR constraints even after employing online adaptation. Of note, patients with pancreatic tumors were treated to the GTV to minimize toxicity risk, which subsequent studies have shown this method may increase locoregional failures.^49^ For other disease sites, delivering BED_10_ ≥100 may not be necessary to achieve promising clinical outcomes. Nevertheless, dose escalation beyond what is typically achievable under CT guidance was possible with MR‐radiotherapy systems. Our results add to the growing body of literature that dose escalation to targets in the abdomen and pelvis are associated with improved clinical outcomes such as LC.

In our cohort, 71of 510 (13.9%) of fractions were adapted. This proportion is lower compared to a prospective trial of SMART for abdominal and pelvic tumors in which 81 of 97 (83.5%) of fractions were adapted.^19^ There are several potential reasons for this discrepancy. The time‐dependent utilization for adaptation could have contributed to a lower proportion of adapted treatments. The majority of adapted fractions occurred later in this study period when adaptive capabilities and workflow were implemented in 2017. Even after implementing a workflow, deciding to trigger adaptation was initially based on visual review of daily volumetric MRI images which we have since learned is not reliable in determining the necessity for adaptation.^27^ The clinical significance of dosimetric violations to OAR or target during predictive plan assessment could have also been considered by managing physicians. It is possible that a dosimetric violation was not considered clinically significant compared to the time and resources re‐optimizing an adapted plan would require, which has reported to take a median of 24 min.^19^, ^26^ This amount of time has potential to introduce additional intra‐fractional motion and increases on‐table treatment time for patients. From early adaptive workflow experiences using the MR‐Cobalt system, the total time to deliver adaptive gated SBRT (from patient entering the treatment room to completing beam delivery) has reported to take up to 90 min, with approximately 90% of patients completing all treatments.^17^, ^19^, ^26^ This duration has improved since implementing iterative workflow process improvements and upgrading to an MR‐LINAC system.^19^, ^28^ More recent experiences have reported delivering adaptive gated SBRT in a median of 45–53 min.^22^, ^28^, ^50^ Finally, physicians often use SMART for improved tumor visualization and localization without intending to utilize online ART. Therefore, opportunities to trigger treatment adaptation may have been missed.

Despite the low rate of adaption utilized in this clinical experience, severe toxicity rates were overall minimal. Contributory factors may include improved soft‐tissue imaging allowing for better target and OAR delineation, as well as breath‐hold gating allowing for smaller PTV margins. As such, the benefits of ART may be more advantageous for specific anatomic sites.

Abdominal tumors, most notably the pancreas, were the most common sites requiring adaptation.^18^, ^19^ In a phase one SMART trial, up to 88% of non‐liver abdominal fractions were adapted to revise OAR constraint violations compared to 36% of fractions directed to liver tumors.^19^ Thresholds to trigger adaptive replanning are currently patient‐ and institution‐specific. Opportunities for more frequent predictive plan adjustments and adaptation may occur with improvements in automatic contouring, automatic plan re‐optimization, more accurate point‐dose accumulation calculations, and adaptive workflow improvements. Our institution has since commissioned an MR‐LINAC which provide additional opportunities to implement ART since more conformal doses can be generated quickly compared to the tri‐cobalt‐60 platform.^24^, ^51^ Altogether, future technological improvements could deliver higher doses for non‐liver tumors, facilitate patient compliance, and reduce treatment time and cost.^52^, ^53^

There are additional limitations. This investigation is retrospective in nature containing a select and heterogeneous population of tumors. However, this analysis was performed on prospectively gathered data of the first 106 consecutive patients which may offset inherent biases associated with many retrospective series. Evaluating clinical outcomes of SMART by every radiation treatment setting, tumor locations, histologies, and BED was not possible because of small resultant sub‐group sample sizes and the likely difficulty in arriving at reliable results. Treatments were performed using 0.35 T MRI. Images generated with a higher magnetic field strength yield higher quality images compared to a lower field strength, which could improve upon auto‐contouring, target localization, and gating. However, there are trade‐offs with using a higher field strength and its overall improvement upon treatment delivery are being characterized at this time. In one report, images from a 0.35T were considered an improvement over cone beam CT scans and sufficient for online ART.^18^ A standard balanced steady‐state free precession (bSSFP) sequence was used for all treatments. New image sequences have been integrated with the MR‐LINAC and additional sequences are under development.

Future prospective trials confirming clinical benefits with SMART by tumor location would be warranted. Additionally, since dose escalation (particularly to BED_10_ ≥ 100) has been associated with clinical benefits from emerging literature, the feasibility of safe dose escalation beyond current dose prescription on MR‐LINAC with daily adaptive planning would be of high interest.

In conclusion, this study demonstrates that SMART permitted safe delivery of ablative radiation for primary and metastatic tumors in the abdomen and pelvis, which corresponded with favorable LC and PFS. Larger prospective trials are needed to confirm these findings.

## CONFLICT OF INTEREST

MC: ViewRay, Inc.: personal fees. JL: ViewRay, Inc: grants, personal fees. NA: Varian, Inc.: personal fees; Brainlab: grants, personal fees, and non‐financial support. DL: ViewRay, Inc: co‐principal investigator of trial, consultant. AR: ViewRay, Inc: grants, personal fees. MS: ViewRay, Inc: personal fees. PL: ViewRay, Inc: clinical advisory board, honorarium, non‐financial support, co‐principal investigator of trial; Varian, Inc: consultant, honorarium, non‐financial support; AstraZeneca, Inc.: research grant, honorarium, non‐financial support. All other authors have no conflict of interest.

## ETHICAL STATEMENT

This study was approved by the institutional review board. Due to the retrospective nature of this study and minimal risk involved to participants, a waiver of informed consent was granted.

## DATA SHARING STATEMENT

The study data are stored in an institutional repository and will be shared upon request to the corresponding author.
